# The prevalence of sensory changes in post-COVID syndrome: A systematic review and meta-analysis

**DOI:** 10.3389/fmed.2022.980253

**Published:** 2022-08-25

**Authors:** Mike Trott, Robin Driscoll, Shahina Pardhan

**Affiliations:** ^1^Vision and Eye Research Institute, Anglia Ruskin University, Cambridge, United Kingdom; ^2^Centre for Public Health, Queen’s University Belfast, Belfast, United Kingdom

**Keywords:** smell, taste, smell loss, taste loss, COVID-19

## Abstract

Post-COVID syndrome can be defined as symptoms of COVID-19 that persist for longer than 12 weeks, with several studies reporting persistent symptoms relating to the sensory organs (eyes, ears, and nose). The aim of this systematic review was to examine the prevalence of persistent anosmia, hyposmia, ageusia, and hypogeusia, as well as eye/vision and ear/hearing related long-COVID symptoms. Authors searched the electronic databases from inception to November 2021. Search terms included words related to long-COVID, smell, taste, eyes/vision, and ears/hearing, with all observational study designs being included. A random effects meta-analysis was undertaken, calculating the prevalence proportions of anosmia, hyposmia, ageusia, and hypogeusia, respectively. From the initial pool, 21 studies met the inclusion criteria (total *n* 4,707; median *n* per study 125; median age = 49.8; median percentage female = 59.2%) and 14 were included in the meta-analysis The prevalence of anosmia was 12.2% (95% CI 7.7–16.6%), hyposmia 29.9% (95% CI 19.9–40%), ageusia 11.7% (95% CI 6.1–17.3%), and hypogeusia 31.2% (95% 16.4–46.1%). Several eye/vision and ear/hearing symptoms were also reported. Considering that changes in the sensory organs are associated with decreases in quality of life, future research should examine the etiology behind the persistent symptoms.

**Systematic review registration:**

[www.crd.york.ac.uk/prospero], identifier [CRD42021292804].

## Introduction

In March 2020, the World Health Organization (WHO) declared the COVID-19 outbreak a global pandemic, and as of March 2022, over 435,500,000 cases have been identified, including over 5,900,000 reported deaths (1). Symptoms of COVID-19 are wide ranging, with commonly reported symptoms including fever, cough, and fatigue (2), although it has also been reported that a significant proportion of infected people are asymptomatic (3, 4). Other well reported symptoms of COVID-19 include anosmia (complete loss of smell), hyposmia (reduced sense of smell), ageusia (complete loss of taste), and hypogeusia (reduced sense of taste), with a loss or changed sense of taste or smell being added to the United Kingdom National Health Service (NHS) official COVID-19 symptoms list in May 2020 (5). Several systematic reviews have identified these sensory symptoms as being highly prevalent in the acute phase of COVID-19. For example, one meta-analysis reported that anosmia or hyposmia are very strong predictors of COVID-19, with a reported risk ratio of 4.6 (6), with another meta-analysis reporting that 50% of COVID-19 patients reported ageusia or dysgeusia in the acute phase (7). COVID-19 symptoms have also been reported in eyes and ears. For example, a recent meta-analysis reported the presence of ocular manifestations of COVID-19 at 11%, with the most common manifestations being dry eye (or foreign body sensation), redness, and tearing (8). Furthermore, COVID-19 has been shown to affect the eyes indirectly, with recent systematic reviews highlighting the negative effect of increases in screen time on eyes, including digital eye strain in both adults and children (9–12). With regards to hearing, a meta-analysis have reported 3% of patients with hearing loss and 4.5% with tinnitus following COVID-19 infection (13).

Post-COVID syndrome (also referred to as “long-COVID”) has been defined as symptoms of COVID-19 that persists for longer than 12 weeks (14, 15). Systematic reviews have identified taste and smell disturbances as symptoms of long-COVID, with respective prevalence rates being reported as 13.5% and 15.2% (16). Another study found the prevalence rate of anosmia > 90 days from COVID-19 infection to be 11%, and ageusia 10% (17). Furthermore, several studies have reported eye related long-COVID symptoms, including blurring (18, 19) and red eyes (20), and also ear related symptoms including hearing loss and tinnitus (21).

One limitation of these reviews is that anosmia and hyposmia (and ageusia and hypogeusia) have not been stratified, therefore the prevalence of graded loss of smell or taste is unknown. Furthermore, current reviews do not appear to have explicitly included studies where patients had no signs of smell or taste disorders prior to COVID-19 infection, introducing an important potential bias. Furthermore, although several reviews have included the long-term effects of COVID-19 involving eyes and ears (16, 22), the wide variations of follow-up make results difficult to compare.

The aim of this systematic review was therefore to examine the prevalence of persistent anosmia, hyposmia, ageusia, hypogeusia, as well as eye and ear related symptoms related long-COVID, across all populations, that persisted for longer than 12 weeks. This research has the potential to inform practitioners, enabling them to monitor possible long-COVID related symptoms, allowing them to potentially create targeted therapeutical and/or mental health management and/or interventions. It will also highlight any need for further primary research studies.

## Methods

The protocol was registered in PROSPERO (protocol number: CRD42021292804). Some changes were made to the protocol, which have been outlined and justified in Supplementary Table 1. Ethical clearance was not required as no primary data was being collected. Two authors (MT, RD) searched the electronic databases PubMed, Embase, Web of Science, and Scopus from inception to November 2021. Search terms included words related to long-COVID, smell, taste, eyes and ears (Full search terms are shown in Supplementary Table 2). In addition, the reference lists of eligible articles were searched for additional articles.

All observational study designs were eligible providing they met all the following inclusion criteria:

1. Studies that reported the prevalence of anosmia, hyposmia, ageusia, hypogeusia symptoms for longer than 12 weeks after COVID-19 diagnosis or studies that reported the prevalence of visual/eye or hearing/ear related symptoms for longer than 12 weeks after COVID-19 diagnosis.
2. Studies written in English.
3. Peer reviewed articles.
4. Participants had no evidence of respective smell/taste/visual/ear related symptoms prior to COVID-19 infection.
5. Reviews were included to manually search reference lists for additional eligible articles.

Title, abstract, and full-text screening was performed independently by two reviewers (MT, RD), and any disagreement between reviewers was resolved by a third senior reviewer (SP).

### Data extraction

Data extraction was completed for each study by two independent researchers (MT, RD) for: (1) author details; (2) year of publication; (3) journal; (4) study type; (5) respective inclusion criteria; (6) demographic information; (7) respective method of data collection; (8) follow-up time (or time elapsed from COVID-19 diagnosis); (9) prevalence data.

### Meta-analysis

For the prevalence of anosmia, hyposmia, ageuisia, and hypogeusia data, a random-effects meta-analysis was conducted using the DerSimonian and Laird method, with studies weighted according the inverse variance, using SPSS Version 28 (23). The meta-analysis was conducted using the following steps:

(1) Prevalence proportions, with associated SEs were inputted, stratified by anosmia, hyposmia, ageusia, and hypogeusia.
(2) Heterogeneity between studies was assessed using the I^2^ statistic (24).
(3) Publication bias was assessed with a visual inspection of funnel plots and with the Egger bias test (25).

Because the data involving ear and eye symptoms was highly heterogeneous (there was not more than one study measuring the same type of eye/ear symptoms, and due to the paucity of studies), there was not enough data to meta-analyze the prevalence rates of these symptoms. The literature regarding eye and ear symptoms, therefore, was synthesized in a narrative analysis.

### Certainty of evidence

To ascertain the certainty of the evidence, the Grading of Recommendations, Assessment, Development and Evaluations (GRADE) framework (26) was used.

### Risk of bias

Risk of bias was assessed by two independent researchers (MT, RD) with the Newcastle Ottawa Scale (NOS) adapted for cross-sectional studies (27, 28). The NOS is a widely used tool and has well established content validity and inter-rater reliability. The NOS has a scoring system based on positive answers to questions regarding: appropriateness of research design; recruitment strategy; response rate; representativeness of sample; objectivity/reliability of outcome determination; power calculation; and appropriate statistical analyses, with points being assigned to positive answers, with a maximum quality score of 10, with higher scores indicating higher quality studies.

## Results

The initial search yielded 5,344 results, of which 2,700 were automatically removed, leaving 2,644 articles that were screened using the titles and abstracts. Of these, 195 articles were selected for full-text retrieval and assessed for eligibility. After full-text assessment, 19 extra articles were found from the reference lists and were further assessed for eligibility. Finally, 21 studies (total *n* 4,707; median *n* per study 125; median age = 49.8; median percentage female = 59.2%) met the inclusion criteria and were included in the review, 14 of which were related to amsonia, hyposmia, ageusia, and hypogeusia [19, 20, 30; 31; 32; 33; 34; 35; 36; 37; 38; 39; 40; 41]. The remaining 10 studies were related to eye and/or ear symptoms (18–21, 29–34) met the inclusion criteria and were included in the narrative analysis.

**FIGURE 1:**
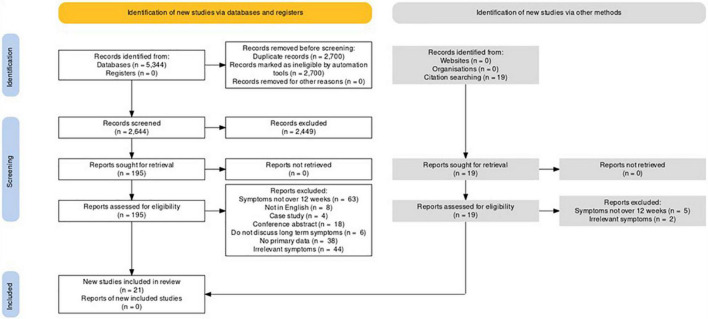
PRISMA flowchart of included studies.

The full PRISMA diagram is shown in Figure 1. Full descriptive characteristics are shown in Table 1. The mean NOS score was 5.4 (median 5; range 4–8)—full NOS scoring can be found in the Supplementary Table 3.

**TABLE 1:** Descriptive characteristics of included studies.

Authors	Study type	Country	Inclusion/exclusion	N	Age	Percentage female	Method of data collection	Symptom duration (type of symptoms)
Bellan et al. (54)	Cohort	Italy	Consecutive patients (or their caregivers) aged 18 years or older who were discharged between March 1 and June 29, 2020, where they had been admitted for COVID-19.	238	Median 61 (IQR 50–71)	NR	Online questionnaire (self-report)	4 months (Anosmia)
Bertlich et al. (52)	Cross-sectional	Germany	All patients that had tested positive for SARS CoV- 2 by polymerase chain reaction.	17	NR	NR	Online questionnaire (self-report)	6 months (Anosmia)
Cristillo et al. (30)	Cross-sectional	NR	People who were hospitalized for mild to moderate (not defined) COVID-19.	101	63.62 (12.9)	27.70%	Self-report–not specified	> 6 months (Eyes/vision)
Davis et al. (21)	Cohort	International	People who had a COVID-19 infection for longer than 1 week	NR	NR	NR	Online questionnaire (self-report)	4–7 months (Eye/vision and ears/hearing)
Garrigues et al. (55)	Cross-sectional	France	People who were admitted to hospital	120	63.2 (15.7)	84.3	Telephone questionnaire	Mean 110.9 days (Anosmia)
Gold et al. (31)	Longitudinal	NR	People who had tested positive for COVID-19 at least 90 days prior to being enrolled	185	43.8 (13.4)	76.7	Self-report–not specified	> 90 days (Ears/hearing)
Gonzalez-Hermosillo et al. (29)	Cohort	Mexico	Consecutive adult patients hospitalized with moderate to severe confirmed COVID-19 pneumonia at hospital admission.	130	51.0 (14)	34.6	Self-report (not otherwise specified)	6 months (Anosmia and ageusia; Eyes/vision)
Hopkins et al. (56)	Longitudinal	United Kingdom	People who reported loss of smell at COVID-19 onset.	434	Median 40 (range 19–77)	74.90%	Online questionnaire (self-report)	6 months (Hypogeusia)
Kim et al. (57)	Longitudinal	Korea	Patients diagnosed with COVID-19.	900	Median 30.5 (IQR 24–47)	69.7	Online questionnaire (self-report)	> 6 months (Anosmia and ageusia)
Klein et al. (32)	Longitudinal	Israel	Israeli residents aged at least 18 years with positive COVID-19 real-time (RT) PCR results. Exclusion criteria were asymptomatic patients and patients with severe COVID-19 infection, defined as receiving respiratory support or intensive care unit admission.	99	NR	NR	Telephone questionnaire (self-report)	6 months (Eye/vision and ears/hearing)
Kumar et al. (33)	Longitudinal	Pakistan	Patients who recovered from COVID-19 and were discharged	817	NR	37.33	Self-structured questionnaire (self-report)	90 days (Eyes/vision)
Lechien et al. (58)	Cross-sectional	18 European countries (not specified)	Ambulatory and hospitalized patients with laboratory-confirmed diagnosis of COVID-19 (nasal swabs-RT-PCR).	233	46 (14.3)	66.1	Objective (Sniffin sticks test)	6 months (Anosmia and hyposmia)
Lemhöfer et al. (59)	Cross-sectional	Germany	NR	365	49.8 (16.9)	59.2	Letter questionnaire (self-report)	3 months (Hypogeusia and hyposmia)
Leth et al. (60)	Longitudinal	Denmark	Patients aged 18 years or older with a positive test who had been hospitalized for at least 12 h and who had been transferred from the emergency department to the Department of Infectious Diseases.	49	Median 58 (IQR 43–78)	57%	Semi-structured interview (self-report)	> 12 weeks (Hypogeusia and hyposmia)
Orru et al. (20)	Cross-sectional	Italy	Adults > 18	152	NR	NR	Online questionnaire (self-report)	3 months (Ageusia, anosmia, eye/vision and ears/hearing)
Osmanov et al. (34)	Longitudinal	Russia	Children (≤ 18 years old) admitted with suspected or confirmed Covid-19.	518	Median 10.4 (IQR 3–15.2)	52.1	Telephone interview (parental-report)	> 5 months (Eyes/vision)
Pilotto et al. (18)	Longitudinal	Italy	Patients who survived COVID-19 disease and were discharged between February and April 2020 from a COVID-19 Unit	31	63.2 (11.2)	32.2%	Structured questionnaire (self-report)	6 months (Eyes/vision)
Rass et al. (19)	Cohort	Austria	Inclusion criteria consisted of (i) confirmed SARS-CoV-2 infection, (ii) hospitalization or outpatient management, and (iii) age ≥ 18 years.	32	Median 45 (IQR 35–55)	69%	Objective smell and taste tests	3 months (Hyposmia, hypogeusia, anosmia; eyes/vision)
Riestra-Ayora et al. (61)	Case control	Spain	The inclusion criteria of the study were: Signed written informed consent for participation in the study; Over 18 years of age; COVID-19 related symptoms; PCR performed from nasal-pharyngeal swab for SARS-CoV-2; Hospital worker at the time of diagnosis; At least 6 months of follow-up The exclusion criteria were as follows: History of nasal surgery, sinusitis, chronic rhinitis, polyposis, olfaction, and/or gustatory disorders; Neurodegenerative disease; A medical or psychological condition that limits the study	100	41.62 (18–65)	80%	Self-report not otherwise specified	> 6 months (Hyposmia, hypogeusia, anosmia)
Taboada et al. (62)	Cross-sectional	Spain	Critically ill patients with COVID-19-induced ARDS admitted to the ICUs hospitals.	91	NR	NR	NR	6 months (Anosmia)
Zhu et al. (63)	Longitudinal	China	Discharged patients clinically diagnosed with COVID-19.	95	49.22 (14.74)	48.4	Hyposmia Rating Scale (self-report)	> 14 weeks (Anosmia)

### Meta-analysis results: Smell and taste

**FIGURE 2:**
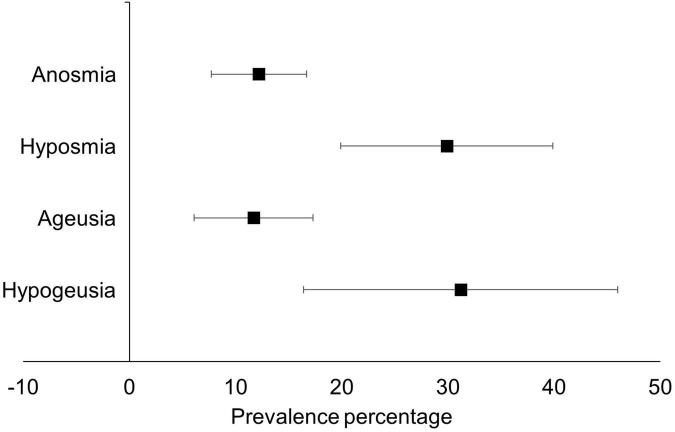
Meta-analytic prevalence of smell and taste related sensory loss in post-COVID syndrome.

Regarding changes in smell, the prevalence of anosmia in people > 12 weeks post-COVID-19 infection was 12.2% (95% CI 7.7–16.6%), and the prevalence of hyposmia was 29.9% (95% CI 19.9–40%). With taste, the prevalence of ageusia was 11.7% (95% CI 6.1–17.3%), with the prevalence of hypogeusia being higher at 31.2% (95% 16.4–46.1%). Full details are shown in Table 2 and Figure 2. The high heterogeneity (which ranged from 89 to 96.8%) and the study designs of included studies, meant that the quality of evidence (according to the GRADE criteria) was classified as low.

**TABLE 2:** Meta-analysis results.

Impairment type	N studies (k outcomes)	Prevalence (95% CI)	I^2^	Eggers bias *P*-value
Anosmia (complete loss of smell)	11 (12)	12.2 (7.7–16.6)	94.4%	0.343
Hyposmia (reduced sense of smell)	8 (8)	29.9 (19.9–40)	93.4%	0.081
Ageusia (complete loss of taste)	4 (5)	11.7 (6.1–17.3)	89.0%	0.781
Hypogeusia (reduced sense of taste)	5 (5)	31.2 (16.4–46.1)	96.8%	0.453

### Persistent eye symptoms

Eight studies (18–21, 29, 30, 32, 34) examined long term visual/eye disorders, all but one of which were in adults, and all were self-reported. The prevalence of persistent blurred vision varied widely across five studies. One study reported 38.5% and 33.1% of participants (the inclusion criteria for participants in this study was not detailed) had an “inability to focus vision” at 3 and 6 months, respectively (29). Cristillo et al. (30) and Pilotto et al. (18), respectively, reported 21.8% and 19.5% prevalence of blurring/loss of vision 6 months after hospital discharge for COVID-19, while Rass et al. (19) reported 7% of participants with blurred vision 3 months after hospital discharge. In children, Osmanov et al. (34) reported a much lower prevalence of 2.1% with “problems seeing/blurred vision” > 5 months post hospital discharge.

Further exploration/analysis from one study (29) showed that prevalence rates of blurred vision or eye symptoms were significantly associated with higher rates of blurred vision in participants reporting fatigue at 3 months post COVID-19 (this was non-significant at 6-months post COVID-19). The study also found that blurred vision prevalence was lower in the “non-fatigue” group from months 3–6 post COVID-19, but not in the “fatigue” sub-group.

Other eye symptoms have been reported. Gonzalez-Hermosillo et al. (29) reported light sensitivity in 18.5% and 20% of participants after 3- and 6-months respectively. Light sensitivity was also associated with fatigue status with significantly higher rates in participants experiencing fatigue compared to those who did 3 months post COVID-19 (this was non-significant at 6-months post COVID-19). Red eyes were also reported in 15.8% of participants 3 months post COVID-19 (20). Two studies also reported non-specific eye and vision related symptoms, including 1% of participants with “eye disorders” 6 months post COVID-19 (32), 23.8% and 26.5% of participants with “vision symptoms” (21) and 2%–13.2% of participants with “other eye symptoms” 4–7 months post COVID-19.

### Persistent hearing/ear disorders

Five eligible studies (19–21, 31, 32) reported long term hearing/ear disorders in adults, all of which were self-reported. Hearing loss was reported in 5.2–6.4% of people between months 4–7 post infection (21). The prevalence of tinnitus varied widely (7–26%) between three studies, with one study reporting rates of between 25 and 26% 4–7 months post infection (21), 14.5% 3 months post infection (20) and 6.9% > 90 days post-COVID infection (31). Vertigo was reported across two studies (19, 20), with prevalence rates ranging from 6 to 13% 3 months post-COVID-19 infection. Furthermore, Rass et al. (19) also found that the prevalence of vertigo was not influenced by the severity of COVID-19 infection. Other studies also reported ear pain and (undefined) “hearing disorder” in 1% of respondents (32), ear ache in 9.9% of respondents (20). Davis et al. (21) reported a prevalence of 8–9% of “other ear/hearing issues” between 4 and 7 months post COVID-19 infection.

## Discussion

This systematic review and meta-analysis pooled the prevalence of anosmia, hyposmia, ageusia, and hypogeusia in people with post-COVID syndrome, and reported on the prevalence of eye and ear related symptoms.

The pooled prevalence of anosmia was 12.2%, which is similar to other systematic reviews (11%) that have measured anosmia in people with COVID symptoms lasting more than 90 days (17). The prevalence of ageusia in our study is also similar to other meta-analyses (11.7% versus 10%). A novel finding in this review was the prevalence of hyposmia and hypogeusia, both of which were much higher than anosmia and hyposmia respectively. A review by Ahmed et al. (35) suggested five possible mechanisms behind anosmia in the acute phase of COVID-19, including the affection of the ACE-2 receptors, damage of supporting cells of the olfactory epithelium, affection of the frontal lobe, inflammatory obstruction of olfactory clefts, and zinc deficiency. In particular, several studies have reported long term damage of the olfactory epithelium in COVID-19 patients (36–38), with some authors recommending specific olfactory theory to improve long-term olfactory function (36). Other authors also suggest a genetic component, indicating that the locus of genes UGT2A1 and UGT2A2 may be a casual factor of anosmia (39). Although it is feasible that these hypotheses could also apply to long-COVID, currently the literature to support this is sparse. Further studies are warranted to ascertain the mechanisms behind smell and taste dysfunction in long-COVID.

Smell and taste dysfunction has been consistency linked with decreases in quality of life (40, 41), as has the presence of long-COVID (42). Furthermore, recent studies have found significant decreases in health-related quality of life in people with long-COVID syndrome (43), with one meta-analysis reporting that 58% of people with long-COVID suffering from poor quality of life (42). This research has the potential to inform practitioners, enabling them to monitor possible long-COVID related symptoms, allowing them to potentially create targeted therapeutical and/or mental health management and/or interventions. It also highlights the need for further primary research studies.

Regarding eye related symptoms, blurring and inability to focus vision ranged from 7 to 39% in adults > 3 months post-COVID-19, with children showing lower prevalence, however, more studies are needed to confirm this. The wide range of prevalence rates is most likely due to the high heterogeneity in various parameters between studies. For example, the inclusion criteria in two of the studies was not specific, indicating possible population bias. It is also not clear, in all of the studies, as to the exact wording of questions to ascertain the presence of blurred vision. On the other hand, it is clear, that participants are self-reporting blurred vision as a persistent post-COVID symptom. The association with fatigue suggests that the blurred vision is possibly due to ciliary muscle’s inability to focus properly, rather than the involvement of other structures of the eye, although this needs to be examined further. The fact that dry eye is also a long COVID symptom suggest a lack of tear production, which would also lead to blurred vision. Detailed clinical measurements should be able to make this clearer. Increased screen time (both leisure and non-leisure) due to the COVID-19 pandemic (11, 12) would exaggerate blurred vision and dry eyes. Signs of viral infection in the eye due to coronavirus, have been reported (44, 45), with studies reporting associations between light sensitivity and chronic fatigue (46), and with digital eye strain (47). The persistent light sensitivity reported here suggest evidence of slow recovery from the infection. Studies have also reported associations between red eyes, fatigue (48) and COVID-19. Further research is warranted to determine the exact causes and etiology of persistent eye symptoms post-COVID-19.

Regarding ear related symptoms, the prevalence of tinnitus varied from 7 to 26% across the studies, also indicating high heterogeneity across studies. Reasons for this heterogeneity could be lack of standardization of sampling techniques and ambiguity around the methods of specific data collection. While exact mechanisms of hearing related disorders in post-COVID syndrome are unknown and warrant further research, it is possible that vascular damage caused by vasculitis, shown as a symptom of COVID-19 (49, 50) may contribute, although more work is needed. It has also been suggested that hearing loss and tinnitus may be associated with other systemic infections not associated with COVID-19 (51), this requires further work.

The findings of this review should be considered within its limitations. Firstly, there was high heterogeneity in our results, which were likely caused by heterogeneous methods of, respectively, measuring anosmia, hyposmia, ageusia, and hypogeusia. In addition, the majority of data were self-reported. Future studies should aim to use homogeneous tools, and wherever possible, objective tests. Secondly, although we only included studies that stated that patients did not have any respective sensory symptoms prior to COVID-19 infection, only one study (52) included a non-COVID-19 control group. It is therefore recommended that future studies be conducted with control groups so reliable prevalence rates can be determined. Third, there were a paucity of studies found regarding eye and ear symptoms, so a meta-analysis was not possible. Fourth, meta-analyses have inherent limitations: their findings are dependent on estimates selected from each primary study and thus are dependent on the accuracy of primary studies (53). Lastly, systematic reviews in rapidly evolving fields such as this one should be considered within the time frame of the systematic literature search. Several articles are published after searches are conducted, and thus the information provided in reviews such has this should be considered as a broad overview of historical information, rather than up-to-date real-time data.

## Conclusion

While anosmia and ageusia appear to be present in around 12% of people 12 weeks post COVID-19, the prevalence of hyposmia and hypogeusia appears to be much higher, with prevalence rates being 30% and 31% respectively. Considering that changes in taste, smell, vision, and hearing are associated with decreases in quality of life and also reduced overall well-being, future research is required to ascertain the mechanisms behind this phenomenon and the creation of therapeutic interventions.

## Data availability statement

Publicly available datasets were analyzed in this study. This data can be found here: All data in this manuscript was retrieved from already existing journal articles. Full information is available from the corresponding author.

## Author contributions

MT: conceptualization, data collection, data analysis, and writing. RD: data collection, data analysis, and writing. SP: conceptualization, writing, and supervision. All authors contributed to the article and approved the submitted version.
