# Supplementary material for: The prevalence of sensory changes in post-COVID syndrome: A systematic review and meta-analysis
Source: Front Med (Lausanne). 2022 Aug 25;9:980253. doi: 10.3389/fmed.2022.980253 (PMC9452774; doi:10.3389/fmed.2022.980253)
Supplement: Supplementary file 1 [file Table_1.DOCX]

Supplementary Table 1: Changes to pre-registered protocol

| Change | Justification |
| --- | --- |
| The method of measuring risk of bias was changed from the JBI tool to the NOS. | With the inclusion of several different study types, more than one JBI tool would have needed to have been used, therefore we changed the risk of bias tool to a validated tool for all observational studies. |

Supplementary Table 2: Full search terms

| Database | Search terms |
| --- | --- |
| Pubmed | (((LONG[Title/Abstract] OR LONG-TERM[Title/Abstract] OR post-acute[Title/Abstract] OR 'late sequela'[Title/Abstract] OR chronic[Title/Abstract] OR long-tail[Title/Abstract] OR persistent[Title/Abstract] OR 'long term'[Title/Abstract]) AND (COVID-19[Title/Abstract] OR COVID[Title/Abstract] OR SARS-CoV-2[Title/Abstract])) OR ('post COVID syndrome'[Title/Abstract] OR 'post-covid syndrome'[Title/Abstract])) AND (eye[Title/Abstract] OR vision[Title/Abstract] OR visual*[Title/Abstract] OR sight[Title/Abstract] OR nose[Title/Abstract] OR smell[Title/Abstract] OR anosmia[Title/Abstract] OR blind*[Title/Abstract] OR hearing[Title/Abstract] OR deaf[Title/Abstract] OR taste[Title/Abstract] OR sense[Title/Abstract] OR sensory[Title/Abstract] OR touch[Title/Abstract]) |
| Scopus | TITLE-ABS-KEY ( ( ( ( ( long OR long-term OR post-acute OR 'late AND sequela' OR chronic OR long-tail OR persistent OR 'long AND term' ) AND ( covid-19 OR covid OR sars-cov-2 ) ) OR ( 'post AND covid AND syndrome' OR 'post-covid AND syndrome' ) ) AND ( eye OR vision OR visual* OR sight OR nose OR smell OR anosmia OR blind* OR hearing OR deaf OR taste OR sense OR sensory OR touch ) ) ) |
| Embase | (((LONG or LONG-TERM or post-acute or 'late sequela' or chronic or long-tail or persistent or 'long term') and (COVID-19 or COVID or SARS-CoV-2)) or ('post COVID syndrome' or 'post-covid syndrome')).mp. and (eye or vision or visual* or sight or nose or smell or anosmia or blind* or hearing or deaf or taste or sense or sensory or touch).ab,kf,ti. |
| Web of Science | (((LONG or LONG-TERM or post-acute or "late sequela" or chronic or long-tail or persistent or "long term") and (COVID-19 or COVID or SARS-CoV-2)) or ("post COVID syndrome" or "post-covid syndrome")) and (eye or vision or visual* or sight or nose or smell or anosmia or blind* or hearing or deaf or taste or senses OR sensory or touch) |

Supplementary Table 3: Full NOS scoring

| **Authors** | **Selection** | | | | **Comparability** | **Outcome** | | **Total score** |
| --- | --- | --- | --- | --- | --- | --- | --- | --- |
|  | Representativeness of the sample | Sample size | Non-respondents | Ascertainment of the exposure | The subjects in different outcome groups are comparable, based on the study design or analysis. Confounding factors are controlled. | Assessment of the outcome | Statistical test |  |
| Cristillo et al. | 1 | 0 | 0 | 1 | 1 | 1 | 1 | 5 |
| Davis et al. | 1 | 0 | 1 | 1 | 1 | 1 | 1 | 6 |
| Gold et al. | 1 | 0 | 0 | 1 | 1 | 1 | 1 | 5 |
| Gonzalez-Hermosillo et al. | 1 | 0 | 1 | 1 | 1 | 1 | 1 | 6 |
| Klein et al. | 1 | 0 | 1 | 1 | 1 | 1 | 1 | 6 |
| Kumar et al. | 1 | 0 | 0 | 1 | 1 | 1 | 1 | 5 |
| Orru et al. | 1 | 0 | 0 | 1 | 1 | 1 | 1 | 5 |
| Osmanov et al. | 1 | 0 | 0 | 1 | 1 | 1 | 1 | 5 |
| Pilotto et al. | 1 | 0 | 1 | 1 | 1 | 1 | 1 | 6 |
| Rass et al. | 1 | 0 | 0 | 1 | 1 | 1 | 1 | 5 |
| Bellan et al. | 1 | 0 | 0 | 0 | 1 | 2 | 1 | 5 |
| Bertlich et al. | 1 | 0 | 0 | 2 | 1 | 2 | 1 | 7 |
| Garrigues et al. | 1 | 0 | 0 | 0 | 1 | 1 | 1 | 4 |
| Gonzalez-Hermosillo et al. | 1 | 0 | 0 | 0 | 1 | 1 | 1 | 4 |
| Hopkins et al. | 0 | 0 | 1 | 0 | 1 | 1 | 1 | 4 |
| Kim et al. | 0 | 0 | 1 | 0 | 1 | 1 | 1 | 4 |
| Lechien et al. | 1 | 0 | 0 | 2 | 1 | 2 | 1 | 7 |
| Lemhöfer et al. | 1 | 0 | 1 | 1 | 1 | 1 | 1 | 6 |
| Leth et al. | 1 | 0 | 1 | 1 | 1 | 1 | 1 | 6 |
| Orru et al. | 1 | 0 | 0 | 0 | 1 | 1 | 1 | 4 |
| Rass et al. | 1 | 0 | 1 | 2 | 1 | 2 | 1 | 8 |
| Riestra-Ayora et al. | 1 | 0 | 0 | 2 | 1 | 1 | 1 | 6 |
| Taboada et al. | 1 | 0 | 0 | 0 | 1 | 1 | 1 | 4 |
| Zhu et al. | 1 | 0 | 0 | 2 | 1 | 1 | 1 | 6 |
